# Pattern of breast cancer presentation during the coronavirus disease pandemic: results from a cohort study in the UK

**DOI:** 10.2217/fon-2021-0970

**Published:** 2022-01-10

**Authors:** Kim Borsky, Ketan Shah, Giles Cunnick, Fiona Tsang-Wright

**Affiliations:** ^1^Bucks Breast Unit, Buckinghamshire Healthcare NHS Trust, High Wycombe, HP11 2TT, UK

**Keywords:** 2-week-wait, breast cancer, COVID-19, metastasis, node, screening, staging, tumor, UICC

## Abstract

**Background:** This study aimed to explore the hypothesis that the stage of breast cancer at initial diagnosis in 2020 is more advanced compared with 2019. **Methods:** Tumor, node, metastasis and Union for International Cancer Control (UICC) stages of new breast cancer diagnoses at the Bucks Breast Unit from May to October 2019 and 2020 were reviewed. A p < 0.05 was considered significant. **Results:** Average UICC stage increased from 1a in 2019 to 2a in 2020 (p < 0.01). Excluding cancers detected through screening, UICC stage still increased from 1b in 2019 to 2a in 2020 (p = 0.0184). There was a significant increase in the percentage of node-positive patients (p = 0.0063) and patients with metastatic disease (p = 0.0295) on initial presentation. **Conclusion:** Overall, patients presented with higher UICC stages and more node-positive and metastatic disease on initial diagnosis in 2020 compared with 2019.

## Lay abstract

During the coronavirus disease 2019 pandemic, breast cancer screening services were halted across the UK. Patients were also encouraged to stay home and to seek medical attention only in an emergency. The authors hypothesized that this might have led to delays in presentation to breast cancer clinics or missed cancer diagnoses. While patients are at home with undiagnosed breast cancer, the cancer can grow and spread. The authors evaluated whether these delays in presentation led to patients presenting with more advanced breast cancers when they finally presented to a breast cancer clinic. The authors collected data on breast cancer stages for a patient group in 2020 (during the height of the pandemic) and compared them with a patient group in 2019. The authors' results did indeed show that patients presented, on average, with more advanced breast cancers in 2020 compared with 2019.

Approximately 55,000 women are diagnosed with breast cancer each year in the UK, less than 15% of whom are locally advanced (stages 3 and 4) on initial presentation [1]. Breast cancer is primarily diagnosed through two pathways: a patient either presents with symptoms such as a palpable lump, skin changes and nipple discharge or a suspicious finding is detected on screening mammogram and consequently investigated. The coronavirus disease 2019 (COVID-19) pandemic has dramatically impacted both pathways of detecting breast cancer. On March 23, 2020, the first national lockdown was imposed across the UK, and the government advised all citizens to stay home unless in need of urgent medical attention. This understandably led to reduced presentation to primary healthcare providers and, consequently, reduced referrals through the 2-week-wait pathway. Furthermore, before the pandemic, breast screening in England was usually offered to women between the ages of 50 and 70 every 3 years [2]. In accordance with the lockdown rules, however, mammographic screening programs were temporarily suspended across the UK in March 2020, a decision that was recommended by several national and international associations for breast surgery and radiology [3,4]. This was mainly in an attempt to reduce footfall in hospitals. With the two main pathways leading to the detection of breast cancer either entirely halted or significantly reduced, a delay in diagnosing new breast cancers was inevitable. Several studies in the past tried to estimate stage progression over time for breast cancer patients but had poor overall accuracy, with reported times for doubling of tumor size varying from 42 to 260 days [5,6]. Although several studies looked into the effects of adapted cancer services during the pandemic, there are limited data specific to breast cancer. A group from Italy estimated that the effects of the suspension of breast cancer screening services led to an increase in undetected breast cancers of 3.43–11.73% [7]. To the best of the authors' knowledge, no study to date has analyzed the changed pattern of presentation in the UK during the pandemic. Based on the delay in presentation due to impaired referral pathways during the pandemic, the authors therefore hypothesized that the stage of presentation at initial diagnosis in 2020 was more advanced compared with 2019.

## Methods

This single-center retrospective study analyzed all new breast cancer diagnoses from May to October 2019 and 2020. Following the suspension of mammographic screening programs across the UK in March 2020 and a period of reorganization of breast cancer services throughout April, the authors evaluated the data starting in May. In the months of May, June and July 2020, mammographic screening was completely suspended. In the three following months, screening restarted, although at reduced capacity, before going back to almost normal capacity in November 2020. To capture the effects of both suspended and reduced screening programs, the authors evaluated the 6 months from May to October 2020 and compared them with the same months in 2019.

Tumor, node, metastasis stage at initial presentation was determined using clinical, radiological and pathological data from the electronic patient records according to the eighth edition of the American Joint Committee on Cancer's Cancer Staging Manual [8]. For all patients treated with surgery, the pathological stage was used; for all others, imaging data were used to determine the tumor stage. Similarly, pathological data were used to determine the nodal status for patients with axillary surgery. Patients who did not undergo axillary surgery had their nodal status derived from imaging. In cases where neither imaging nor surgical data were available, clinical data were used. Likewise, metastatic status was primarily derived from staging images.

Node-positive patients and patients with recurrence or clinical suspicion of metastatic disease were staged with CT images of the chest, abdomen and pelvis and whole body bone scans. Patients who did not qualify for staging (first breast cancer, node-negative) and had no clinical suspicion of metastatic disease on examination of commonly affected regions (lung, abdomen, spine, pelvis and neurological exam) were considered free of distant metastases (M0). For patients receiving neoadjuvant chemotherapy, the clinical stage at presentation was used rather than the pathological stage, as neoadjuvant chemotherapy led to considerable downstaging and the focus of interest was the stage at initial presentation. Only patients who did not have sufficient data for tumor, node, metastasis classification or had recurring disease were excluded (both homo- and contra-lateral recurrences). Union for International Cancer Control (UICC) combined stage was determined from tumor, node, metastasis classification. The initial stage was compared for the cohorts as a whole and grouped according to the mode of detection (referred for symptoms vs detected through screening), as screening on average detects less advanced stages.

All statistical analyses were performed using R 4.0.2 (R Foundation for Statistical Computing, Vienna, Austria). The *t*-test was used for continuous data, and chi-square test was used to analyze categorical data. Wilcoxon rank sum test was performed to determine a significant shift in UICC stage between the two cohorts at initial presentation. For all analyses, a p < 0.05 was considered significant.

## Results

A total of 472 patients had a new breast cancer diagnosis during the data acquisition period. A total of 439 patients met the inclusion criteria, and 33 were excluded. Key parameters and patient characteristics are summarized in Table 1. A total of 276 patients were diagnosed in 2019, and 163 were diagnosed in 2020. The difference in total new diagnoses was primarily due to the significant decrease in screening activity during the first 3 months analyzed in 2020. Although 121 (43.8%) patients had a screen-detected cancer in 2019, this number dropped to 15 (9.2%) in 2020. In 2019, a little more than half (56.2%) of the patients were diagnosed through referrals compared with 90.8% in 2020. The median age at initial diagnosis was comparable at 63 (interquartile range: 54–72) in 2019 and 62.5 (interquartile range: 50–74) in 2020.

**Table 1.** Patient characteristics for both groups and p-values indicating significant differences.

Patient characteristics	2019	2020	p-value
n	276	163	
Age, median (IQR)	63.0 (54–72)	62.5 (50–74)	0.1226
Detection, n (%) Referral Screening	155 (56.2) 121 (43.8)	148 (90.8) 15 (9.2)	<0.0001
Node-positive on presentation, n (%) No Yes	211 (76.4) 65 (23.6)	104 (63.8) 59 (36.2)	0.0063
Metastatic on presentation, n (%) No Yes	268 (97.1) 8 (2.9)	150 (92.0) 13 (8.0)	0.0295
Median UICC stage at detection Overall Referral Screening	1a 1b 1a	2a 2a 2a	<0.0001 0.0184 0.0445

IQR: Interquartile range; UICC: Union for International Cancer Control.

A total of 65 (23.6%) patients presented with node-positive disease in 2019, and 59 (36.2%) presented with node-positive disease in 2020 (*X*^2^ = 7.47; p = 0.0063). A total of eight (2.9%) patients had metastatic disease on presentation in 2019, and 13 (8%) had metastatic disease on presentation in 2020 (*X*^2^ = 4.74; p = 0.0295).

**Figure 1.**
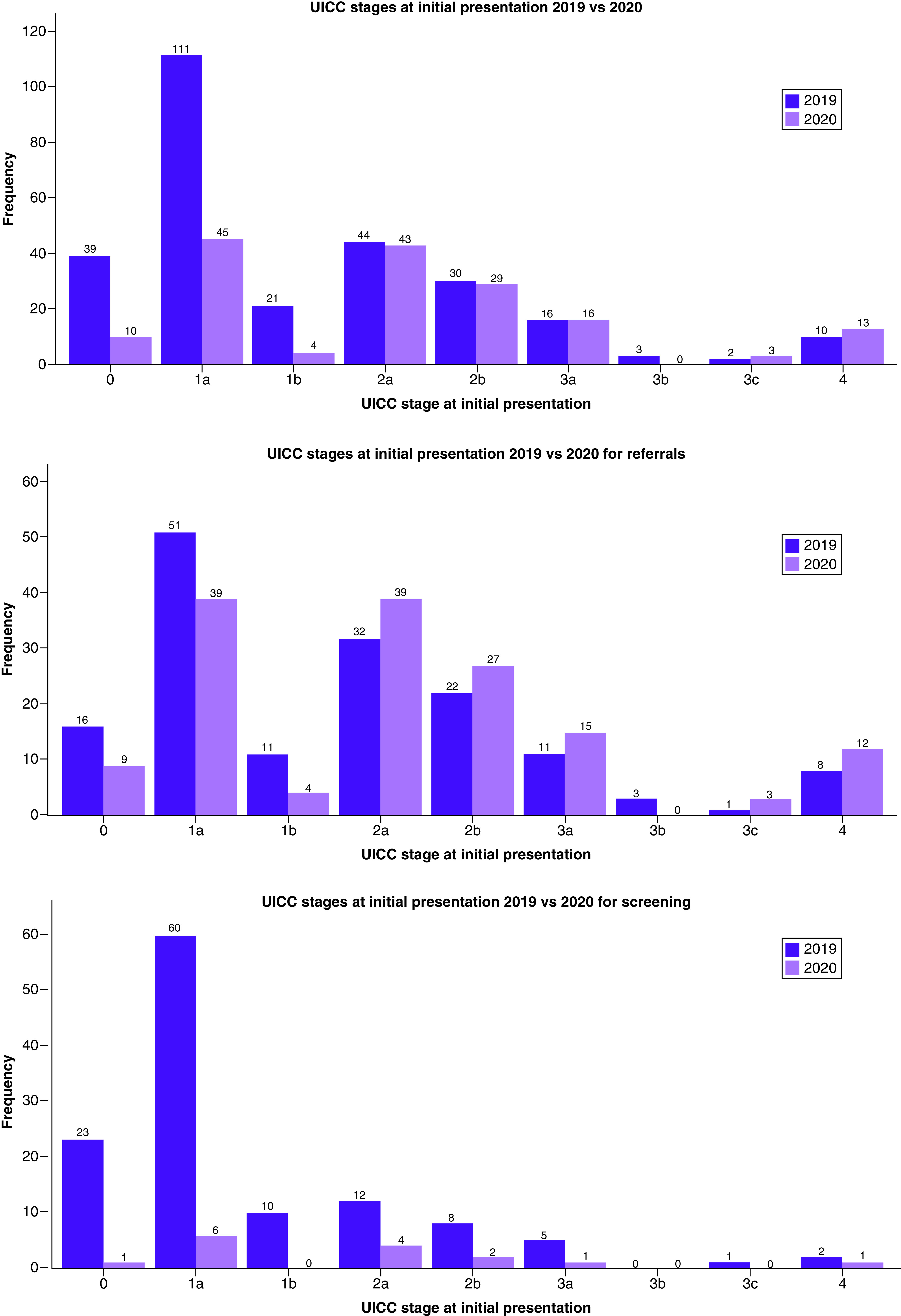
Union for International Cancer Control stages at initial presentation in 2019 versus 2020. Overall results (top) and results for diagnoses made through referrals (middle) and screening (bottom), respectively, are shown. UICC: Union for International Cancer Control.

As shown in Figure 1, 31 (11.2%) patients presented with stage ≥3 cancer in 2019, and 32 (19.6%) presented with stage ≥3 cancer in 2020. When comparing only diagnoses through referrals, 23 (14.8%) patients presented with stage ≥3 cancer in 2019, and 30 (20.3%) presented with stage ≥3 cancer in 2020. For diagnoses made through screening, eight (6.6%) patients had stage ≥3 cancer in 2019, and two (13.3%) had stage ≥3 cancer in 2020.

Median UICC stage at initial presentation was 1a in 2019 and 2a in 2020 (W = 16,564; p < 0.0001). When comparing only diagnoses through referrals, the median UICC stages were 1b and 2a, respectively (W = 9713; p = 0.0184). With regard to diagnoses made through screening mammograms, the median UICC stage was 1a in 2019 and 2a in 2020 (W = 636; p = 0.0445).

## Discussion

### Interpretation

The authors' findings show that the overall UICC stage at initial presentation significantly increased during the COVID-19 pandemic. This is particularly noteworthy considering that in 2019 the proportion of patients with locally advanced stages (≥3) was 10.9%, which is well below the reported national average of 15% [9]. Furthermore, this almost doubled in 2020 to 19.6%. This can intuitively be explained by the interruption in screening programs, which, on average, detect smaller, asymptomatic and node-negative breast cancers [10–12]. With the temporary pause in screening, it seems logical that, on average, the UICC stage would increase in the remaining patients diagnosed with breast cancer. Interestingly, there was also a significant increase in UICC stage when comparing only patients diagnosed through symptomatic referrals. This may be a reflection of the government's advice to stay home during the first national lockdown as well as the adapted working patterns of primary care providers. A patient's hesitation in seeking medical advice or difficulties getting an appointment with a primary care provider and an appropriate referral could have led to their presenting later to breast clinics.

Both node-positive breast cancer and metastatic disease on initial presentation also significantly increased during the pandemic. This is of particular interest, as these two factors can significantly influence the management of breast cancer. If chemotherapy or radiotherapy is required to treat such advanced cancers adequately, this in turn increases footfall to hospitals and puts added pressure on cancer services. However, one of the main rationales behind pausing the screening programs was to reduce the number of patients visiting hospitals and free up capacity for COVID-related tasks.

These findings are largely in keeping with a similar study performed by Toss *et al.* in a breast cancer center in Italy [13]. In their single-center analysis comparing breast cancer stages during a trimester in 2019 with a trimester in 2020, when screening programs were interrupted, the researchers found a significant decrease in *in situ* disease as well as a significant increase in node-positive and stage 3 disease. Similarly, Oldani *et al.* predicted more advanced stages of breast cancer due to the national lockdown and temporarily interrupted screening in Italy [7].

Although not directly evaluating a change in stages at presentation, Peacock *et al.* noted a 56% decline in breast cancer diagnoses in Belgium during the pandemic [14]. Scioscia *et al.* estimated the number of missed breast cancer diagnoses in Italy due to the interruption in screening programs at 2793 [15], and in Spain, breast cancer diagnoses showed a 26.1% decrease in 2020 [16]. These numbers show the profound impact of the pandemic on oncological services and help to identify patients at risk. To minimize the negative impact of the pandemic, post-pandemic protocols should focus on targeting patients at risk.

### Strengths & limitations

As a retrospective analysis, this study has limitations inherent in its design. Furthermore, the patient cohort is from a single breast unit in a geographic area where the predominant ethnicity is white British and the economic status is above average. To increase the applicability of the aforementioned findings to the general population in the UK, a multicenter study would therefore be advisable. A multicenter design could also address the limited patient numbers, especially with regard to the screening population in 2020. Although this study found a statistically significant increase in median UICC stage when comparing only diagnoses through screening, numbers in the 2020 subgroup were very limited (n = 17), and therefore it remains doubtful whether this finding is also of clinical significance. By contrast, the single-center study design, with a limited number of clinicians assessing images and clinical status for staging, reduces inter-rater variability and allows for homogeneous methodology and data acquisition.

### Present & future research

The findings of advanced stages of breast cancer on initial presentation need to be put in the context of potential long-term consequences. Sharpless modeled national cancer data on colorectal and breast cancer for the USA to predict excess deaths from altered screening, diagnosis and treatment [17]. His conservative predictions estimate a little more than 5000 excess deaths from breast cancer by 2030. Similarly, Yong *et al.* used mathematical modeling to predict advanced cancer stages and cancer deaths in Canada following breast cancer screening interruptions of different lengths [18]. The researchers found that a 3-month interruption in screening could lead to 310 more advanced-stage diagnoses and 110 additional deaths from cancer.

Sud *et al.* specifically looked at the reduction in 2-week-wait referrals and its implications for breast cancer patients in England [19]. In their study, in which they modeled a 3-month lockdown leading to an average delay in presentation of 2 months, they found that 181 additional lives and 3316 life-years would be lost based on a conservative backlog estimate of 25%. These numbers increased to 542 additional lives and 9948 life-years lost for a 75% backlog. Similarly, Maringe *et al.* modeled cancer deaths in England caused by delays in diagnosis due to the pandemic [20]. For breast cancer, they estimated between 281 and 344 (7.9–9.6%) additional deaths up to year 5 after diagnosis.

These projections clearly highlight the importance of reinitiation of screening programs and implementation of robust strategies to cope with the backlog to avoid additional deaths from delays in detection. Future work should closely monitor the practice in the UK during the recovery phase of the pandemic with an aim to return to pre-COVID service provision nationwide as soon as possible. This is currently being investigated by the B-MaP-C study [21].

## Conclusion

Delays in the 2-week-wait pathway and the temporary interruption of screening programs led to increased stages at initial presentation of breast cancer patients as well as an increase in the proportion of node-positive and metastatic disease on diagnosis. Although several modeling studies predict additional cancer deaths due to this stage shift, there is a lack of literature auditing the current practice in the recovery phase of the pandemic in the UK. In order to derive recommendations for optimizing cancer care and to minimize the negative long-term effects of the altered pathways implemented during the pandemic, this should be the focus of current research.

## Future perspective

The main focus for the next few years has to be the return of cancer service provision to pre-COVID standards. Screening programs are largely running at full capacity again nationwide but there is a massive backlog to clear. Furthermore, the ‘stay-at-home' mentality of the past 2 years is still ingrained in patients mind and this issue will have to be addressed in order to prevent cancer progression due to late presentation.

**Summary points**

- Union for International Cancer Control stage at presentation in 2020 was significantly higher than that observed in 2019.
- Union for International Cancer Control stage at presentation in 2020 was significantly higher in patients who were referred for symptoms than that observed in 2019.
- Union for International Cancer Control stage at presentation in 2020 was significantly higher in patients who were screened than that observed in 2019.
- The proportion of patients presenting with node-positive disease in 2020 was significantly higher than that observed in 2019.
- The proportion of patients presenting with metastatic disease in 2020 was significantly higher than that observed in 2019.
